# BMI and mortality: the limits of epidemiological evidence

**DOI:** 10.1016/S0140-6736(16)30949-7

**Published:** 2016-07-13

**Authors:** David Berrigan, Richard P Troiano, Barry I Graubard

**Affiliations:** Division of Cancer Control and Population Sciences (DB, RPT) and Division of Cancer Epidemiology and Genetics (BIG), National Cancer Institute, National Institutes of Health, Bethesda, MD 20892, USA

Concurrent with the global increase in obesity,^1^ numerous studies and reviews have been published concerning associations of overweight and obesity with mortality. Their findings have prompted considerable public health debate. There is ongoing discussion as to whether cutoff points for body-mass index (BMI) categories should differ across regions or racial or ethnic groups.^2^ Additionally, studies differ in their assessment of the relation between BMI and mortality. In particular, BMI in the overweight category (BMI 25–<30 kg/m^2^) is not consistently associated with increased mortality.^3,4^ To improve precision, observational studies have been combined in pooled analyses and meta-analyses. Pooled analyses benefit from the use of harmonised methods but, unlike meta-analyses, are not generally based on systematic review.

In *The Lancet*, The Global BMI Mortality Collaboration^5^ presents results from the largest ever pooled dataset about the relation between BMI and mortality. Their data began with 239 studies, in 32 countries, with more than 10 million participants and about 1·6 million deaths. Prespecified analyses of never smokers without pre-existing chronic disease, excluding the first 5 years of follow-up, are presented for about 3·9 million participants and about 386 000 deaths occurring in 189 studies. This study is notable for the use of standardised methods to extract hazard ratios (HRs) for mortality across the studies and for extensive and valuable appendices that examine diverse subsets of the pooled data. The authors made efforts to address reverse causation and residual confounding. Results are broadly similar to other recent studies.^3,6,7^ For example, HRs were J-shaped, with increased risk of mortality for both low BMI and obesity (BMI ≥ 30 kg/m^2^). This study documents heterogeneity in the association between BMI and mortality across different continents and shows weaker associations in older populations, especially in those aged 70 years and older. For overweight adults (BMI 25–<30 kg/m^2^), HRs—adjusting for age and sex and excluding individuals with baseline chronic disease—ranged from 0·99 (95% CI 0·98–1·00) after adjustment for smoking to 1·11 (1·10–1·11) after exclusion of ever smokers and the first 5 years of follow-up. The elevated HR of 1·11 in overweight adults after exclusion of about 60% of the sample and about 75% of deaths is a key result of this pooled analysis.

Two major issues are raised by this important paper. The first is whether conclusions about the relation between BMI and mortality from analyses with extensive exclusions can be generalisable and unbiased. The second is what sort of public health guidance can be obtained from analyses that pool global data. Substantial research and conceptual questions remain for each of these issues. Samples of different distributions of environmental and person-specific factors (eg, disease history, diet, and physical activity) are difficult to address consistently in large pooling studies. Exclusion of ever smokers, deaths in the first 5 years of follow-up, and pre-existing chronic disease (where available) might further increase these differences. Extensive exclusions might also limit the generalisability of resulting health recommendations. Selection bias and early mortality exclusion, to control for potential bias from weight change due to occult disease leading to reverse causation, pose additional challenges for inference from pooling studies.^8^ For example, Monte Carlo simulation and analytical analyses of the association between BMI and mortality indicate that exclusion of 2 years or 5 years of early mortality does not necessarily reduce bias and can even increase bias of estimated HRs.^9^


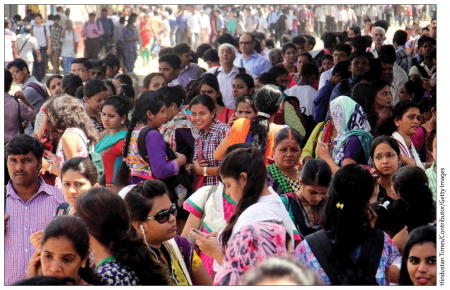


Challenges in deriving global public health recommendations are unlikely to be resolved by ever-larger datasets without further developments in study data and design. New study designs such as mendelian randomisation,^10^ new data elements such as weight histories,^11^ and increased attention to BMI over the life course,^12^ might improve our understanding of the links between excess bodyweight and mortality. The present study compares data from four continents, pooling data across diverse racial and ethnic groups, and across countries with very different patterns of chronic disease management.^2^ Large studies of specific race and ethnic groups, such as that of Yi and colleagues of 12·8 million Koreans,^4^ can help to clarify recommendations for specific countries or demographic groups even if these studies cannot conclusively address the limits of observational studies.

Despite the limitations of observational studies for causal inference of obesity and mortality,^13^ many crucial questions about BMI will continue to rely on observational data. To date, few sufficiently sized randomised trials have been done to address whether weight-loss interventions reduce mortality or morbidity. One trial^14^ was ended after about 10 years of follow-up because no association between weight loss and cardiovascular events was found. Weight-loss interventions have only modest long-term effectiveness^15^ and generally target behaviours, such as diet and physical activity,^13^ that can lead to change in BMI rather than directly targeting BMI itself. Therefore, clinical trials are limited in their capacity to address causal relations between BMI and mortality.

Important challenges remain in the effort to translate epidemiological evidence of excess bodyweight and mortality into effective guidelines and public health interventions. *The Lancet*, via the World Obesity Federation, and other coalitions such as the US National Collaborative on Childhood Obesity Research, are championing diverse approaches to this challenge including support for better measurement, systems models, and increased attention to the evaluation of obesity-related policies.
